# Reliability and validity of assessing energy and nutrient intake with the Vienna food record: a cross-over randomised study

**DOI:** 10.1186/s12937-019-0431-9

**Published:** 2019-01-28

**Authors:** Peter Putz, Birgit Kogler, Isabel Bersenkowitsch

**Affiliations:** 1 FH Campus Wien – University of Applied Sciences, Favoritenstraße 226, 110 Vienna, Austria; 2 Present Address: Scientific Staff, FH JOANNEUM – University of Applied Sciences, Alte Poststraße 149, 8020 Graz, Austria

**Keywords:** Test-retest reliability, Concurrent validity, Prospective food record, Weighed food record, Energy and nutrient intake

## Abstract

**Background:**

The Vienna Food Record was developed as a simple paper-based pre-coded food record for use in Austrian adults, which can be completed over a flexible period of time. The present study aimed at evaluating test-retest reliability of the Vienna Food Record and its concurrent validity against a weighed food record*.*

**Methods:**

A randomised cross-over study served to compare outcomes of the Vienna Food Record with those of the weighed food record. The Vienna Food Record was completed for a second time, in order to assess test-retest reliability. Three assessment phases were interrupted by two-week wash-out phases*.* Sixty-seven free living Austrians aged 18–64 years, without (self-) diagnosed food allergies or intolerances, not at any medication, and not nutrition experts, were randomly assigned to one of two study arms. After drop-outs and exclusion of under-reporters, data of 35 participants has been analysed. Paired t-tests were performed for comparisons, regarding test-retest reliability and criterion validity, where mean differences were calculated as effect sizes. Consistency between repeated assessments with the Vienna Food Record was expressed by intra-class-correlation coefficients (ICC), while Pearson’s r was used for agreement regarding validity. Bland-Altman Plots with 95% limits of agreement were created for energy and macronutrients. Validity metrics for macronutrients were analysed additionally separated by gender, taking an adjustment of energy intake into account. Total energy intakes as well as intakes of macro- and selected micronutrients, expressed as daily means, were defined as 34 primary outcomes.

**Results:**

ICCs for energy and intake of preselected nutrients, expressing the consistency of the Vienna Food Record, ranged from not significant to 0.95. Pearson’s correlation coefficients, expressing the agreement of the Vienna Food Record with the weighed food record ranged from not significant to 0.80*.*

**Conclusions:**

This study demonstrates acceptable reliability and validity of the Vienna Food Record as an instrument for the assessment of energy and nutrient intake, comparable to the results of similar studies.

## Background

Self-reported dietary intake is assessed by methods of prospective records and methods of recall [1]. Food records are considered an accurate way of dietary intake assessment [2]. However, especially weighed food records (WFR), are very time consuming for participants and the research personnel [3]. Recall methods are, thus, employed more frequently [1]. Amongst those, Food Frequency Questionnaires (FFQ), which evaluate a person’s usual intake over a defined period of time, are relatively cheap and easy to administer [4]. Standards for the evaluation of the intake of food, nutrients, and potentially hazardous chemicals by means of 24-h recalls have been developed and validated within the European Food Consumption Validation (EFCOVAL) project [5]. Self-administered web-based applications of 24-h-recalls gained popularity recently [3, 6, 7]. As compared to food records, recalls have been shown to be more prone to over-reporting low intakes, and under-reporting high intakes, which has also been referred to as the flat slope syndrome [8]. While all of these aforementioned methods underlie potential sources of error, the participants’ inability to fully and accurately recall their intakes, specifically applies to recall methods [1]. Prospective records, on the contrary, may be subject to influence the participants’ dietary behaviour [9]. The Vienna Food Record (VFR) was developed as simple paper-based prospective food record for use in Austrian adults, which can be completed over a flexible period of time, with a minimum of three weekdays and one weekend day being recommended as a minimum to draw conclusions on the overall dietary behaviour. However, the VFR may also be used for assessing a single meal. A likewise prospective food record has been developed and validated for use in the German part of the EPIC (European Prospective Investigation into Cancer and Nutrition) project [10]. The VFR includes a brief introduction page and infographics to support the estimation of portion sizes, and can hence be completed without an interview or instruction by an expert. 182 predefined food items were selected in order to meet specific requirements of Austrian users. Within this user-centred design approach, user feedback on selected food items, clarity and usability was collected throughout three iterations. The VFR has been embedded in the software package nut.s [11]. The software integrated evaluation routine facilitates analysing a completed VFR in about 10 min, by entering the sum of portions for each food item recorded. Moreover, the VFR is freely available for non-commercial teaching and research (Creative Commons CC BY-NC-ND 3.0 AT https://creativecommons.org/licenses/by-nc-nd/3.0/at/deed.en). Details on the development process of the VFR are published elsewhere [Bersenkowitsch I, Kogler B, Tritscher A, Visontai S, Putz P: User-centered development of a prospective estimated dietary record for use in Austrian adults: The Vienna food record, submitted]. The aim of the present study was to evaluate the VFR concerning its test-retest reliability and concurrent validity against a WFR as reference method.

## Methods

### Aim and design

The design of a randomised cross-over study was chosen in order to avoid bias arising from the sequence of protocol completion and to require a smaller sample size [12]. Equal numbers of participants were randomly allocated to a study arm, completing first the VFR and then the WFR, or vice versa, respectively. Finally all participants completed the VFR for a second time, with the purpose of obtaining outcomes concerning test-retest reliability. The examinations were interrupted by two-week wash-out phases in order to reduce the risk of possible carry-over effects related to diet or diet-recording behaviour. No changes to the specifications of materials, methods and outcomes were made, after the study has commenced. The collection of data ended as scheduled after the finalisation of the assessments taking place between February and March 2018.

### Participants and setting

Subjects were considered eligible if they were 1) 18–64 years old, 2) without (self-)diagnosed food allergies or food intolerances, 3) not at any medication, 4) not a nutrition expert (such as nutrition scientists or dietitians) or in education therefor, and 5) provided informed consent. Participants were excluded after randomisation, if they 1) were classified as under-reporters, 2) were classified as over-reporters, 3) reported energy intakes differing more than two standard deviations (SD) between two assessments, 3) commenced with any medication due to sickness, 4) left Austria in the course of an active assessment phase. Participants were classified as under-reporters, if they reported an average daily energy intake smaller than their basal metabolic rate multiplied by 1.1, where basal metabolic rate was estimated with the Henry equations [13], based on self-reported body size and weight. Participants were classified as over-reporters, if they reported an average daily energy intake higher than 4500 kcal. Deliberations on how to critically evaluate energy intake are described elsewhere [14, 15]. All participants were residents of Austria and they were visited at home by a member of the study team to receive instructions and study materials. Material was handed out and instructions were given by students of dietetics in their final year of academic education, who received 3 h of training for that purpose.

**Fig. 1:**
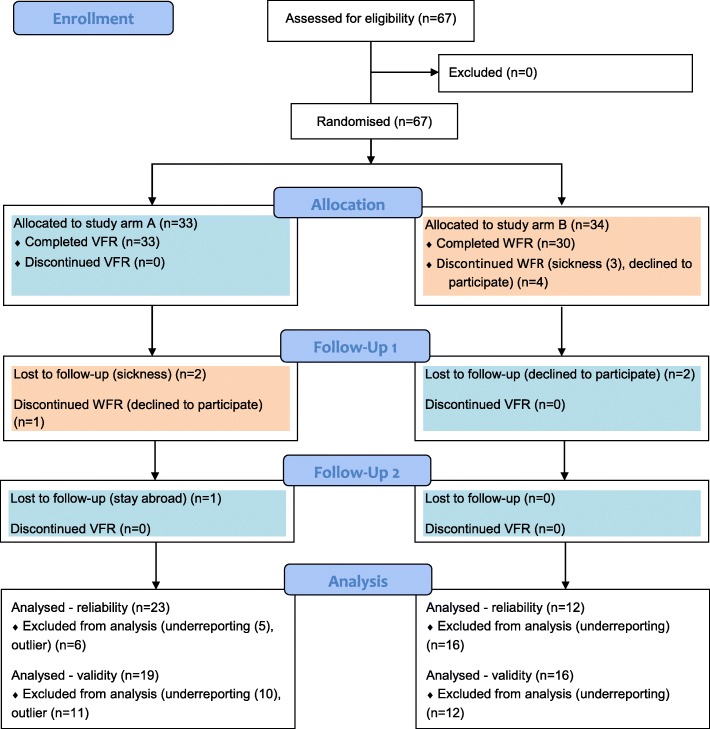
CONSORT flow diagram^21^ showing the progress of participants through the phases of the crossover randomised study. Blue boxes refer to assessments with the Vienna Food Record (VFR), and orange boxes refer to assessments with the weighed food record (WFR). Participants were classified as under-reporters, if self-reported average daily energy intake was smaller than their basal metabolic rate multiplied by 1.1, where basal metabolic rate was estimated with the Henry equations [13]

A sample size of 49 participants proved to be sufficiently statistically powered in a comparable study [16], and hence 50 participants has been aimed for. Considering an estimated drop-out rate of 25%, 67 subjects were enrolled. The principal investigator used an online sequence generator [17], to randomly allocate the participants to one of the two study arms (Fig. 1), without applying any clustering or blocking approaches. All study material (case report forms, protocols, scales, picture books) were labelled with the individual participant code, including an indication on the allocation. Due to the nature of the studied assessments, no measures were taken regarding allocation concealment and participant blinding. Data entry and analysis was done by blinded outcome assessors, where pseudonyms were created by means of participant coding.

### Outcome measures

Each of the three assessments was carried out, in the time between February and March 2018, over four consecutive days, including one weekend day. Participants were instructed to select, and stick with, one out of two options: to record from Wednesday to Saturday, or from Sunday to Wednesday, respectively. For the completion of the VFR participants received no further instructions, but to read the information provided on its cover page and backside. This includes one A5-format page, explaining how to protocol consumed items and one A5-format page, showing infographics supporting the estimation of portion sizes, e.g. 150 g for a full portion of meat. For the completion of the WFR, the aforementioned students of dietetics explained the process of completing the protocol using a pre-filled example and a written guideline. In brief, the guideline instructed 1) to weigh all foods before consumption, as well as leftovers, with a kitchen scale (Soehnle Vita 65,119), 2) how to precisely describe the food item (product specifications where applicable), 3) how to indicate fat content (where applicable) and way of preparation, 4) not to skip drinks or in-between meals, 5) how to indicate out-of-home consumption. Components of mixed dishes were weighed separately. Intake of dietary supplements was recorded and included in the analyses, in both VFR and WFR. Regarding out-of-home consumption, participants were asked to take pictures of foods/dishes with their smartphone and to match their pictures later on with a print-out of the Austrian adaptation of a portion size picture book, provided with friendly permission by the International Agency for the Research on Cancer (IARC). Participants were encouraged to contact the principal investigator in case of uncertainty, and they were offered to receive an overview, compiling the results of their WFR after having all study procedures completed. Apart from that, no incentives were given. Total energy intakes and intakes of macro- and micronutrients, expressed as daily means were defined as 34 primary outcomes, where micronutrients were selected as done for the Austrian Nutrition Report 2017 [18]. Dietary intake data was analysed using *nut.s nutritional software* (https://www.nutritional-software.at) in its recent version (May 2018) [11], based on the German food composition database *Bundeslebensmittelschlüssel* (BLS*)* and its Austrian extension. All VFR were entered into the software by one person, and all WFR were entered by another person. Gender, age, self-indicated body weight, height, and highest completed education were recorded as background information. Moreover, a system usability survey was filled in after completing the VFR twice, as a secondary pre-specified, outcome. Hence, the usability sample is equal to the one described for test-retest reliability. Five questions, in the style of the Food4Me FFQ validation study [16], assessed whether the participants perceived the VFR as 1) easy to complete, 2) time consuming, 3) interesting to complete, 4) subject to make them reflect their dietary behaviour, and 5) something they would be willing to complete again in future. For each question, one box of a closed five-level scale had to be checked, including the options “applies fully”, “applies rather”, “neutral”, “applies rather not” and, “does not apply”.

### Statistical analyses performed

Shapiro-Wilk tests and graphical inspections of histograms were performed to check data for normality. Data were expressed as daily means including SD. Independent t-Tests were performed, to see whether the VFR outcomes of follow-up 2 differed between study arm A and study arm B. For comparisons, regarding test-retest reliability and criterion validity, paired t-tests were performed, and effect sizes were expressed as mean differences, including an indication as percentage. For reliability, the consistency of a test, intra-class-correlation coefficient (ICC) and standard error of measurement (SEM) are described as common metrics [19]. For the test-retest evaluation, ICCs (3.1) were calculated for absolute agreement of single values. SEM (SEM = SD √(1-ICC)) was calculated in order to examine the precision of the measurement in the unit of the specific outcome (e.g. kcal/d), where the standard deviation for all test scores was derived from the total sum of squares of the ICC’s ANOVA (SD = √(SS/(n-1)) [19]. Pearson’s r was used to express agreement for validity. Moreover, Bland-Altman Plots [20] with 95% limits of agreement have been created for energy and macronutrients. Due to the wide range of possible applications of the VFR, no indications for clinically acceptable deviations were defined a priori. Alpha was set at 0.05 – *p*-values rounded to the second position after decimal points are reported throughout the manuscript. Statistical analysis was performed with IBM SPSS Statistics version 24 [21].

## Results

### Summary

An overview on the numbers of participants randomly assigned to a study arm, carried out assessments, and analysed for the primary outcomes is summarised in Fig. 1, by means of a CONSORT flow chart [22]. Participants’ outcomes were analysed for validity, if assessments were complete at baseline and follow-up 1. Reliability analyses were carried out, if the VFR was completed twice. After drop-outs and exclusion of under-reporters, 35 participants remained in the analysis of reliability, and also, largely overlapping, 35 participants in the analysis of validity. None of the participants was classified as over-reporter. One participant was excluded based on initial outlier screening, with more than two SD difference in energy intake between two assessments, both for reliability and validity. For 4 out of 34 observations (protein, cholesterol, iodine, phosphorus), the VFR outcomes in follow-up 2 differed significantly between the two study arms. Based upon the overall consistency, a pooled reliability analysis, merging both study arms, was carried out. For outcomes like Vit D and alcohol, neither the Shapiro-Wilk test nor the graphical inspection supported the assumption of approximate normality. However, due to the high share of participants scoring “0” in these outcomes, parametric options for data presentation and inferential statistics were performed for those and all outcomes.

### Overview of the study population

Baseline demographic characteristics are summarised in Table 1. Men are underrepresented in the sample, and on average younger than female participants. Due to this eventual lack of gender balance, gender separated validity outcomes were analysed additionally for macronutrients, taking an adjustment of energy intake (2500 kcal/d) into account. Body mass indices of all participants analysed, ranged from 17.0–31.1 kg/m^2^, based on self-reported indications of body weight and height. 29% of the participants analysed, indicated a university degree as highest completed education, while 21% did not have a general qualification for university entrance. When comparing participants included in the validity analysis (*n* = 35), with those lost to follow up, excluded as under-reporters or outliers (*n* = 30), no significant deviations were observed regarding, age, BMI, and level of education. Another two participants were randomly assigned to a study arm, but did not report such data. Concerning the analysis of reliability and usability, there were no significant deviations regarding age and level of education. However, BMI differed significantly (*p* = 0.01) between participants analysed (*n* = 35, mean: 22.2, SD: 3.0) and those lost to follow-up and excluded (*n* = 30, mean: 24.3, SD: 3.6).

**Table 1:** Age and BMI^a^ of the study population in total, and separated by gender

Study	n	Demographic characteristics, mean (SD)	
		Age (years)	BMI^a^
Reliability / Usability			
Men	11	29.7 (10.3)	24.3 (3.2)
Women	24	35.9 (12.4)	21.2 (2.4)
All	35	34.0 (12.0)	22.2 (3.0)
Validation			
Men	13	28.5 (10.0)	24.2 (3.0)
Women	22	38.0 (11.2)	21.3 (2.4)
All	35	34.6 (11.6)	22.4 (3.0)

^a^Body mass index (kg/m^2^) based on self-reported indications

### Test-retest reliability of the VFR

Table 2 provides an overview of outcomes related to test-retest reliability. With a mean difference of 56.2 kcal/d (3%), energy intake was fairly equal between the two assessments. Although the outcomes for 2 out of 34 observations (SFA, riboflavin) differed significantly, there were generally consistent mean intakes. On average, there was 7% difference between the test and retest. ICCs ranged from 0.01 to 0.95 (Vit D), with some observations being not significant (PUFA, Vit B6, Vit B12, Vit C, Vit A, retinol equivalents). On average, ICC was 0.52, including values of the aforementioned not significant observations. Next to ICC, Table 2 displays SEM as a further metric of consistency.

**Table 2:** Test-retest reliability of the VFR for daily energy and nutrient intake, *n* = 35

	Test^a^ mean (SD)	Retest^b^ mean (SD)	Mean diff. (%)	p^c^	ICC (3.1)^d^	p^e^	SEM^f^
Energy (kcal)	2245 (496)	2301 (596)	−56.2 (−3)	0.449	0.69	< 0.001	384.9
Total fat (g)	91.3 (27.3)	98.3 (32.6)	−7.0 (−8)	0.135	0.59	< 0.001	24.4
SFA (g)	35.3 (11.8)	39.7 (14.0)	−4.4 (− 12)	0.013	0.67	< 0.001	9.6
MUFA (g)	33.2 (11.8)	35.0 (12.4)	−1.8 (−5)	0.313	0.63	< 0.001	9.3
PUFA (g)	16.7 (7.2)	16.8 (7.5)	−0.1 (− 1)	0.948	0.19	0.136	8.3
Cholesterol (mg)	405 (176)	380 (139)	24.6 (6)	0.375	0.48	0.002	144.3
Protein (g)	95.3 (44.6)	94.3 (33.2)	1.0 (1)	0.868	0.61	< 0.001	30.9
Carbohydrates (g)	244 (48)	239 (59.6)	4.8 (2)	0.616	0.48	0.002	49.1
Fibres (g)	25 (9)	23.3 (8.6)	1.3 (5)	0.322	0.66	< 0.001	6.6
Total sugars (g)	86 (34)	88.5 (31.4)	−2.5 (−3)	0.639	0.54	< 0.001	27.8
Alcohol (g)	7.0 (9.0)	9.4 (11.6)	−2.4 (− 34)	0.251	0.30	0.037	11.0
Calcium (mg)	1002 (423)	1086 (457)	−83.6 (−8)	0.112	0.75	< 0.001	276.1
Chloride (mg)	5924 (1777)	6061 (2271)	− 137 (−2)	0.705	0.46	0.002	1879
Iron (mg)	16.0 (7.0)	15.5 (5.8)	0.4 (3)	0.720	0.37	0.014	6.4
Iodine (μg)	117 (43.8)	116 (45.3)	1.4 (1)	0.851	0.52	0.001	38.8
Magnesium (mg)	425 (151)	412 (147)	12.7 (3)	0.563	0.63	< 0.001	113.9
Phosphorus (mg)	1459 (530)	1504 (528)	−45.1 (−3)	0.415	0.82	< 0.001	286.3
Potassium (mg)	3233 (858)	3517 (1238)	− 284 (−9)	0.105	0.54	< 0.001	916.7
Sodium (mg)	3526 (1120)	3520 (1270)	6.6 (0)	0.971	0.61	< 0.001	942.7
Zinc (mg)	12.9 (5.3)	13.6 (4.7)	−0.7 (−5)	0.344	0.63	< 0.001	3.9
Thiamine (mg)	1.3 (0.4)	1.4 (0.5)	−0.1 (−8)	0.119	0.57	< 0.001	0.4
Riboflavin (mg)	1.7 (0.6)	1.8 (0.7)	−0.2 (−12)	0.007	0.81	< 0.001	0.3
Niacin eq. (mg)	36.9 (13.0)	37.7 (13.8)	−0.8 (−2)	0.691	0.58	< 0.001	10.9
Vit B5 (mg)	4.9 (1.4)	5.2 (1.8)	−0.4 (−8)	0.054	0.76	< 0.001	1.0
Vit B6 (mg)	1.7 (0.7)	1.9 (1.0)	−0.2 (− 12)	0.391	0.18	0.151	1.0
Vit B7 (μg)	54.2 (24.6)	52.3 (19.4)	1.9 (4)	0.651	0.42	0.006	21.3
Folic acid (μg)	291 (88.6)	310 (139)	−19.9 (−7)	0.232	0.65	< 0.001	86.6
Vit B12 (μg)	7.7 (9.0)	6.4 (3.8)	1.3 (17)	0.435	0.01	0.467	8.7
Vit C (mg)	105 (63.4)	135 (201)	−30.5 (−29)	0.367	0.12	0.236	188.7
Vit A - Carotene (μg)	8.0 (5.9)	8.1 (8.5)	−0.1 (−1)	0.914	0.06	0.371	8.9
Retinol eq. (μg)	1.8 (1.0)	1.8 (1.5)	0 (0)	0.959	0.13	0.231	1.5
Vit D (μg)	5.1 (8.9)	4.9 (10.3)	0.2 (4)	0.759	0.95	< 0.001	12.1
Tocopherol eq. (mg)	15.3 (8.3)	13.6 (6.2)	1.7 (11)	0.108	0.64	< 0.001	5.6
Vit K (μg)	234 (162)	277 (317)	−43.0 (−18)	0.366	0.39	0.009	247.5
Average			7%		0.52		

^a,b^Vienna Food Record (VFR), completed over 4 consecutive days including 1 weekend day

^c^2-sided *p*-value derived from paired t-test

^d^Intra-class correlation coefficient (3.1): absolute agreement, single values, two-way mixed

^e^*p*-value derived from ICC

^f^Standard error of measurement

### Agreement of the VFR with the WFR as reference method

**Fig. 2:**
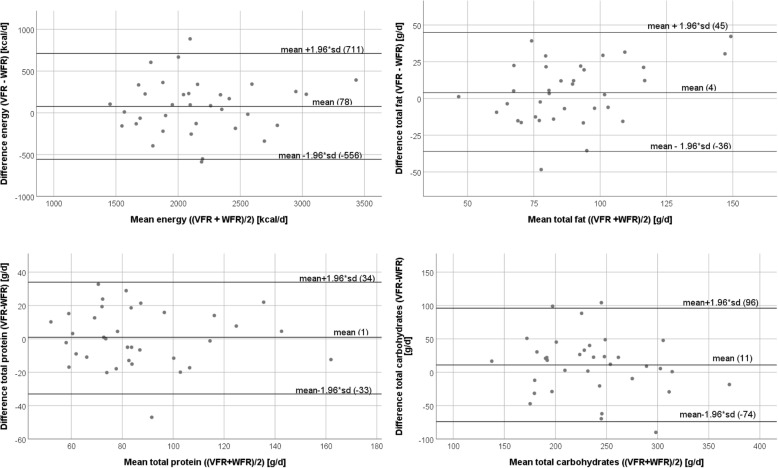
Bland Altman plots for energy, fat, protein and carbohydrate intake, *n* = 35. Lines indicate mean differences and 95% limits of agreement, VFR … Vienna Food Record, WFR … weighed food record

Table 3 shows results related to criterion validity. For 5 out of 34 observations (MUFA, Vit B6, Vit C, Vit A, Vit K), the outcomes differed significantly. On average, there was 9% difference between the VFR and the WFR. Pearson’s correlation coefficients ranged from 0.15–0.80 (protein), with a small number of observations being not significant (cholesterol, Vit A, retinol equvivalents). On average, Pearson’s r was 0.60, including values of not significant observations. Figure 2 shows Bland-Altman plots with 95% limits of agreement, comparing the VFR with the WFR for energy and macronutrients. As for reliability, the energy intake was fairly similar between the two methods, with a mean difference of 78 kcal/d. Proportional bias was observed for fat (beta: 0.39; T: 2.40, p: 0.022), but not for energy, protein, and carbohydrates.

**Table 3:** Concurrent validity of the VFR against a WFR for daily energy and nutrient intake, *n* = 35

	VFR^a^ mean (SD)	WFR^b^ mean (SD)	Mean diff. (%)	p^c^	Pearson’s r	p^d^
Energy (kcal)	2197 (490)	2119 (472)	77.5 (4)	0.165	0.78	< 0.001
Total fat (g)	91.0 (27.4)	86.7 (20.1)	4.3 (5)	0.234	0.66	< 0.001
SFA (g)	35.8 (11.8)	36.2 (9.3)	−0.4 (−1)	0.831	0.55	0.001
MUFA (g)	33.0 (12.0)	28.9 (7.2)	4.0 (12)	0.015	0.62	< 0.001
PUFA (g)	16.2 (7.0)	16.3 (5.6)	−0.1 (−1)	0.966	0.42	0.011
Cholesterol (mg)	364 (149)	371 (150)	−7.2 (− 2)	0.829	0.15	0.401
Protein (g)	87.2 (26.6)	86.5 (27.4)	0.7 (1)	0.811	0.80	< 0.001
Carbohydrates (g)	241 (50.9)	230.1 (58.8)	10.7 (4)	0.154	0.70	< 0.001
Fibres (g)	24.7 (9.0)	22.7 (9.4)	2.0 (8)	0.143	0.63	< 0.001
Total sugars (g)	85.3 (31.2)	90.8 (29.5)	−5.5 (−6)	0.180	0.70	< 0.001
Alcohol (g)	6.9 (8.8)	7.5 (9.6)	−0.6 (−9)	0.722	0.47	0.004
Calcium (mg)	1036 (400)	1052 (466)	−16.4 (−2)	0.759	0.75	< 0.001
Chloride (mg)	5733 (1907)	5316 (1604)	416.4 (7)	0.133	0.60	< 0.001
Iron (mg)	15.3 (6.9)	15.4 (6.9)	−0.1 (−1)	0.854	0.79	< 0.001
Iodine (μg)	112.6 (44.1)	108 (46.9)	4.6 (4)	0.535	0.55	0.001
Magnesium (mg)	418 (149)	400 (132)	18.4 (4)	0.345	0.68	< 0.001
Phosphorus (mg)	1416 (459)	1464 (489)	−48.0 (−3)	0.357	0.80	< 0.001
Potassium (mg)	3166 (802)	2989 (850)	176.4 (6)	0.100	0.72	< 0.001
Sodium (mg)	3386 (1200)	3141 (1062)	245.0 (7)	0.195	0.54	0.001
Zinc (mg)	12.2 (3.6)	11.7 (3.5)	0.5 (4)	0.213	0.76	< 0.001
Thiamine (mg)	1.3 (0.4)	1.3 (0.4)	0.0 (0)	0.485	0.65	< 0.001
Riboflavin (mg)	1.7 (0.6)	1.6 (0.5)	0.0 (0)	0.805	0.66	< 0.001
Niacin eq. (mg)	34.2 (10.3)	36.0 (12.7)	−1.8 (−5)	0.236	0.71	< 0.001
Vit B5 (mg)	4.6 (1.5)	5.0 (1.7)	−0.4 (−9)	0.107	0.67	< 0.001
Vit B6 (mg)	1.7 (0.7)	1.8 (0.8)	−0.2 (−12)	0.085	0.76	< 0.001
Vit B7 (μg)	52.2 (25.6)	59.0 (37.3)	−6.9 (−13)	0.235	0.48	0.004
Folic acid (μg)	289 (94.6)	312 (108)	−23.5 (−8)	0.136	0.61	< 0.001
Vit B12 (μg)	6.5 (7.4)	4.7 (2.2)	1.8 (28)	0.127	0.40	0.017
Vit C (mg)	99.5 (52.2)	170 (191)	−70.3 (−71)	0.023	0.43	0.010
Vit A - Carotene (μg)	7.6 (5.6)	5.2 (3.8)	2.5 (33)	0.021	0.22	0.200
Retinol eq. (μg)	1.7 (1.0)	1.4 (0.8)	0.3 (18)	0.130	0.30	0.082
Vit D (μg)	4.9 (8.9)	6.0 (8.8)	−1.1 (−22)	0.349	0.68	< 0.001
Tocopherol eq. (mg)	15.3 (8.2)	15.5 (8.7)	−0.2 (−1)	0.889	0.48	0.004
Vit K (μg)	222 (160)	158 (153)	64.0 (29)	0.004	0.69	< 0.001
Average			10%		0.60	

^a^Vienna Food Record (VFR), completed over 4 consecutive days including 1 weekend day

^b^Weighed food record (WFR), completed over 4 consecutive days including 1 weekend day

^c^2-sided *p*-value derived from paired t-test

^d^2-sided *p*-value derived from Pearson’s correlation

Table 4 shows exploratory outcomes related to criterion validity for macronutrients as shown in Table 3, but separated by gender. In order to adjust for gender differences in energy intake, these data were divided by the respective energy intake in kcal/d and multiplied by 2500. Agreement was markedly stronger in men (ranging from 0.64–0.87), as compared to women (ranging from 0.26 (not significant) - 0.52).

**Table 4:** Concurrent validity of the Vienna Food Record against a weighed food record for energy adjusted (per 2500 kcal/d) daily macronutrient intake by gender

	VFR^a^ mean (SD)	WFR^b^ mean (SD)	Mean diff. (%)	p^c^	Pearson’s r	p^d^
Men (*n* = 13)						
Fat (g)	153.6 (48.7)	143.6 (29.0)	10.0 (7)	0.36	0.64	0.02
Protein (g)	159.8 (39.8)	153.5 (40.1)	6.3 (4)	0.29	0.87	< 0.01
Carbohydrates (g)	409.8 (64.9)	386.4 (78.8)	23.4 (6)	0.15	0.72	< 0.01
Average			5%		0.74	
Women (*n* = 22)						
Fat (g)	160.5 (39.0)	155.7 (35.3)	4.8 (3)	0.54	0.52	< 0.01
Protein (g)	143.8 (25.4)	145.9 (34.7)	−2.1 (− 2)	0.79	0.26	0.24
Carbohydrates (g)	423.5 (82.6)	407.7 (100.3)	15.7 (4)	0.45	0.45	0.03
Average			3%		0.41	

^a^Vienna Food Record, completed over 4 consecutive days including 1 weekend day

^b^weighed Food Record, completed over 4 consecutive days including 1 weekend day

^c^2-sided *p*-value derived from paired t-test

^d^2-sided *p*-value derived from Pearson’s correlation

### System usability of the VFR

In terms of the VFR’s system usability, 26 of 35 (74%) perceived it as easy or rather easy to complete, 10 of 35 (29%) indicated that it was time consuming or rather time consuming, and 23 of 35 (66%) found it interesting or rather interesting to complete. 26 of 35 (74%) fully or rather agreed that the VFR made them reflect their dietary behaviour. 20 of 35 (57%) fully or rather agreed that they would be willing to complete the VFR again in future. Generally, no important harms or unintended effects have been observed.

## Discussion

### Discussion of the results

The energy intake derived from the VFR appeared to be fairly reproducible over time (ICC = 0.69), strongly correlated to the WFR as reference method (r = 0.78), and generally within a very plausible magnitude (test: mean 2245, sd: 496 kcal/d; retest: mean 2301, sd: 596 kcal/d). In the first VFR, men reported a mean energy intake of 2736 kcal/d (*n* = 11, sd: 433); while women reported a mean intake of 2020 kcal/d (*n* = 24, sd: 338 kcal). A likewise Danish study validating a pre-coded food diary, reported a mean energy intake of 2317 kcal (sd: 573) for both genders [23]. Differences between the two assessments were found for some micronutrients, both for reliability and agreement with the reference method. While ICCs for energy and nutrient intake regarding test-retest reliability of the VFR ranged from 0.01–0.95, Pearson’s correlation coefficients, expressing agreement with the reference method, ranged from 0.15–0.80. The aforementioned Danish study, reported Pearson’s correlation coefficients from 0.16–0.71, in this context [23]. Energy, protein, and carbohydrates were among the outcomes with the highest correlation coefficients, being above 0.7 in both studies. Validation studies on self-administered web-based applications of 24-h-recalls, reported Pearson’s correlation coefficients up to 0.75 [6], and from 0.06–0.64 [7], respectively. The number of outcomes and specifically micronutrients analysed in these studies, were fairly similar. When testing energy adjusted macronutrient intake, validity of the VFR was better in men (average r: 0.74) than in women (average r: 0.41). Supposing that women are able to complete the VFR as accurate and complete as men do, this may be due to a generally higher inconsistency of diet behaviour in women. Although only 57% of the participants fully or rather agreed that they would be willing to complete the VFR again in future, there was generally positive response in terms of system usability.

### Implications for clinical practice

For clinical practice, the VFR may be recommended for estimations of energy and nutrient intake, e.g. as a basis for a dietetic consultation. For the interpretation of repeated assessments from a single client, clinicians may also calculate the so called minimal detectable change (MDC) based on values of SEM provided in Table 2. MDC_95_ indicates the required change of a measurement that would, with a certainty of 95%, exceed the outcome’s test-retest variability (MDC_95_ = SEM × 1.96 x √2) [24]. Health professionals, like dieticians, may also find the additional software output “intake of food groups” useful. As indicated in the VFR introduction page, a minimum of 4 days including one weekend day should serve as basis to draw conclusions on the overall dietary behaviour. The VFR may also serve as an instrument for research studies conducted in Austria, while for cross-national comparisons preference should be given to instruments that were specifically designed for this purpose. Conclusions on the overall dietary behaviour, based upon a 4 day assessment with the VFR, need to be drawn more conservatively for women, as compared to men.

### Strengths and limitations

The randomised cross-over design, with the WFR carried out in a rigid and transparent way, with extensive instructions by trained students of dietetics and making use of the IARC picture book to account for out-of-home consumption, are major strengths of this study. The sample has not been drawn representatively for the general population, and is also small. This introduces limitations regarding the generalisability of findings. A relatively large number of participants needed to be excluded due to under-reporting. Consequently, the results reported, may be applied for users reporting a plausible daily energy intake above their estimated basal metabolic rate multiplied by 1.1. Moreover, possible seasonal changes were not taken into account. Albeit considered the most accurate way for assessing habitual dietary intake, prospective food records may influence users’ behaviour and thus introduce bias [9, 23]. Since nutritional epidemiology is still lacking a gold-standard measurement, such a “validation study” can only aim at understanding the structural equation of the measurement error model rather than to assess the validity of an instrument measuring dietary intakes. Hence, the administration of a combination of both, objective biomarkers and subjective reports is becoming increasingly popular to address methodological limitations, such as comparing one self-reported tool against another [1].

## Conclusions

The VFR is a simple paper-based pre-coded dietary intake record, which is fully flexible regarding the duration of logging. However, the provided details regarding its reliability and validity refer to a period of four consecutive days, including one weekend day. The study resulted in acceptable reliability and agreement with the reference method, with a very plausible estimation of energy intake. These results are comparable to those of similar validation studies of prospective records carried out in other countries.

## Abbreviations

ANOVA: analysis of variance
BLS: *Bundeslebensmittelschlüssel* of the German food composition database
BMI: body mass index
D: day
diff: difference
EFCOVAL: European Food Consumption Validation project
EPIC: European Prospective Investigation into Cancer and Nutrition project
eq: equivalent
FFQ: Food Frequency Questionnaire
g: grams
IARC: International Agency for the Research on Cancer
ICC: intra-class-correlation coefficient
kcal: kilocalories
MDC: minimal detectable change
mg: milligrams
MUFA: mono-unsaturated fatty acid
n: number (of participants)
p: probability (statistical)
PUFA: poly-unsaturated fatty acid
r: Pearson’s correlation coefficient
SD: standard deviation
SEM: standard error of measurement
SFA: saturated fatty acid
VFR: Vienna Food Record
Vit: vitamin
WFR: weighed food record
μg: micrograms
